# Phylodynamic reconstruction of global evolution and geographic structure of maize chlorotic mottle virus

**DOI:** 10.1099/jgv.0.002323

**Published:** 2026-09-02

**Authors:** Jianguo Shen, Yujie Jin, Shayidan Wufuer, Zhenguo Du, Fangluan Gao

**Affiliations:** 1 Fujian Key Laboratory for Technology Research of Inspection and Quarantine, Technology Center of Fuzhou Customs District, Fuzhou 350001, P. R. China; 2 Institute of Plant Virology, Fujian Agriculture and Forestry University, Fuzhou 350002, P. R. China

**Keywords:** cryptic circulation, maize chlorotic mottle virus, MASCOT-Skyline, molecular dating, whole-genome sequence

## Abstract

Maize chlorotic mottle virus (MCMV) represents a major quarantine pathogen that poses a serious threat to global maize production, yet its spatiotemporal evolutionary dynamics remain incompletely characterized. In this study, we reconstructed the global molecular epidemiology of MCMV by analysing 117 complete genome and 214 coat protein (CP) gene sequences. We employed whole-genome data for Bayesian phylodynamic inference while utilizing CP sequences for phylogenetic reconstruction and population genetic analyses. Our phylodynamic analyses estimated a mean evolutionary rate of 2.07×10^−4^ substitutions per site per year, with the global most recent common ancestor traced to the Americas around 1938. Following its emergence, the MCMV population diversified into two major clades: a basal American lineage (Clade I) and a recently emerged, rapidly diversifying lineage (Clade II, originating ~1952). Within Clade II, we identified a monophyletic East African cluster – representing the most extensively sampled geographic population – that is phylogenetically nested within a broader assemblage of Asian isolates. This East African population, dating to the mid-1980s, exhibits signatures of a recent founder effect, characterized by minimal intra-regional genetic differentiation and lower nucleotide diversity (*π*=0.003) relative to Asian (0.008) and American (0.026) populations. Phylodynamic demographic reconstructions reveal that regional establishment in East Africa coincided with a pronounced global expansion in effective population size (*N*_e_) from the mid-1990s through the mid-2000s, subsequently followed by demographic stabilization. These findings provide structural and temporal insights into the global population structure and spatiotemporal dynamics of MCMV, establishing a foundation for international surveillance strategies and phytosanitary control measures.

## Data availability

All data used in this study are publicly available on NCBI through accession number PZ270488. A list of the accession numbers used is found in Table S1. All .xml data files are available on request from the author.

## Introduction

Maize chlorotic mottle virus (MCMV) belongs to the species *Machlomovirus zeae*, the type species of the genus *Machlomovirus* (family *Tombusviridae*) [1]. Initially discovered in Peru in 1973 [2], MCMV subsequently emerged across other distinct geographic regions, including Hawaii, Mexico and Argentina [3–5]. Maize (*Zea mays*) is the only known natural host for MCMV and remains the sole economically important species affected by this pathogen. However, controlled inoculation experiments have demonstrated a broader experimental host range within the family *Poaceae* [6]. The severity of MCMV-associated disease is strongly influenced by maize genotype and developmental stage at the time of infection. Symptoms range from mild chlorotic mottling to severe yellowing, systemic tissue necrosis and premature plant [7]. Infected plants may also exhibit pronounced growth abnormalities, including severe stunting, reduced tassel development with sparse spikelets and the production of undersized, deformed and poorly filled ears. Under field conditions, MCMV infection alone typically results in grain yield losses of 10–15%. Its epidemiology is driven by efficient mechanical transmission and multiple dispersal pathways. The virus is transmitted by insect vectors, such as thrips [8] and several species of chrysomelid beetles [9]. Critically, MCMV can also persist in soil [10] and be transmitted via contaminated seeds [11], facilitating both long-distance spread and environmental persistence. Notably, the impact of MCMV is greatly amplified during synergistic co-infections with potyviruses, leading to maize lethal necrosis (MLN), a highly destructive disease [11]. This synergism further underscores the importance of elucidating the evolutionary dynamics of MCMV.

At the molecular level, the MCMV genome consists of a single-stranded, positive-sense RNA molecule of ∼4.4 kb, which lacks both a 5'-cap structure and a 3'-poly(A) tail [1]. The genomic RNA typically encompasses seven ORFs, encoding proteins essential for viral accumulation (P32), replication (P50 and its readthrough product P111), cell-to-cell movement (p7a and P7b), systemic spread (P31) and encapsidation (coat protein, CP) [12, 13]. Despite its relatively small genome size, MCMV exhibits remarkable evolutionary plasticity, enabling it to adapt to diverse agro-ecological environments across continents. The rapid geographic expansion of MCMV in recent decades, culminating in the emergence of devastating MLN outbreaks in East Africa in 2011, has stimulated the accumulation of viral sequence data and comparative analyses of isolates from Africa, Asia and the Americas [14, 15]. These studies have revealed pronounced geographic structuring of the global MCMV population, including low genetic diversity within East African and Chinese populations and a close phylogenetic relationship between isolates from these regions. Nevertheless, because previous investigations have largely relied on regional comparisons and static phylogenetic reconstructions, the temporal origins of the major lineages, historical changes in population size and pathways of intercontinental spread remain poorly resolved within a unified global evolutionary framework.

To address these gaps, we conducted an integrated molecular epidemiological and phylodynamic analysis using an extensive collection of CP gene sequences and a curated, non-recombinant whole-genome dataset. Our analyses indicated that the global MCMV population most likely originated in the Americas around 1938 and subsequently diversified into two major clades. We further identified a pronounced increase in global effective population size from the mid-1990s to the mid-2000s and evidence of a relatively recent founder event associated with the establishment of MCMV in East Africa. Together, these findings provide a clearer temporal and evolutionary framework for understanding the global emergence and spread of MCMV and may inform international surveillance and phytosanitary control strategies.

## Methods

### Virus isolates and dataset curation

On 8 November 2019, an isolate of MCMV was intercepted from maize seeds (hereafter referred to as MCMV-TH). Total RNAs were extracted from the samples using the E.Z.N.A.^®^ Plant RNA Kit (Omega Bio-tek, Norcross, GA, USA) according to the manufacturer’s instructions. To obtain the complete MCMV genome of this isolate, ten pairs of specific primers (Table S1) were designed based on the conserved regions of published MCMV genomes and used to amplify overlapping fragments by real-time PCR (RT-PCR). Sequences of the 5′ and 3′ ends of the viral genome were amplified by rapid amplification of cDNA ends (RACE) using the SMARTer RACE 5′/3′ Kit (TaKaRa, Dalian, China) following the manufacturer’s instructions. All PCR products were cloned into the pMD18-T vector (TaKaRa, Dalian, China) and at least three independent clones per fragment were sequenced by Sangon Biotech (Shanghai, China). Sequences were aligned and assembled into a consensus genome using DNAMAN v9.0 (Lynnon Biosoft, Quebec, Canada). The complete genome sequence of MCMV-TH was deposited in the GenBank database with the accession number PZ270488.

To facilitate comprehensive evolutionary analyses, two distinct datasets were curated alongside the novel sequence generated in this study. The first, designated as the FG (full-genome) dataset, consisted of 121 complete genome sequences and was employed for recombination screening and the assessment of temporal signals. The second, designated as the CP dataset, comprised 214 CP gene sequences (including the CP region of MCMV-TH) and was utilized for assessing genetic diversity and population differentiation. All reference sequences were retrieved from GenBank using the VirPhyKit facility [16]. Detailed metadata for these isolates, including geographic origins and collection dates, are summarized in Table S2 and Table S3. Multiple sequence alignments were performed using the MAFFT algorithm [17] integrated in the PhyloSuite v2.0 platform [18].

### Recombination analysis

For the FG dataset, we evaluated the potential impact of reticulate evolution using two distinct approaches. First, a split network was constructed using the split decomposition method, and the pairwise homoplasy index (PHI) was calculated via the neighbour-net algorithm in SplitsTree v4.13.1 [19] to screen for global recombination signals. Subsequently, individual recombination breakpoints were characterized using seven heuristic methods (RDP, GENECONV, BOOTSCAN, MAXCHI, CHIMAERA, SISCAN and 3SEQ) within the RDP 5.84 suite [20]. To ensure high stringency and filter out spurious signals, only recombination events detected by at least four independent methods with a Bonferroni-corrected *P*-value ≤0.05 were considered significant. After excluding four isolates (DSMZ PV-1208, DSMZ PV-1087, KS1 and Nebraska) due to incomplete metadata, a refined dataset comprising 117 complete genome sequences was retained for subsequent evolutionary analyses.

### Assessment of phylogenetic temporal signal

We initially evaluated the clock-like behaviour of the FG dataset by regressing root-to-tip divergence against isolation years in TreeTime [21], based on an ML tree (*GTR +I+Γ*_4_) generated via PhyloSuite v2.0 [18] using IQ-TREE v1.6.8 [22]. To further corroborate the temporal signal and rigorously account for potential biases caused by non-uniform temporal sampling, we followed the analytical framework proposed by Murray *et al*. [23]. First, a Mantel test was performed to assess the correlation between pairwise genetic distances and temporal distances, thereby testing for confounding effects between temporal and genetic structures. Subsequently, to mitigate potential Type I errors identified in the standard test, we implemented a cluster-based date-randomization test [24] using BEAST v1.10.4 [25]. In this procedure, sampling dates were systematically shuffled only among distinct phylogenetic clusters rather than across the entire dataset, generating ten randomized replicates. The presence of a robust temporal signal was determined by the lack of overlap between the 95% CI of the substitution rate estimates from the real data and those from the randomized iterations. MCMC chains were run for 5×10^7^ iterations, sampling every 5×10^3^ steps. We discarded the initial 10% as burn-in and confirmed sufficient posterior sampling (effective sample size, ESS>200) and chain convergence through visual diagnostic check.

### Phylodynamics and structured coalescent analysis

To infer migration and population dynamics from the FG dataset, we employed the MASCOT 3.0.7 framework (marginal structured coalescent approximation) [26]. Subpopulations (demes) within the structured population were designated based on the distinct clusters identified in the phylogenetic network analysis. To identify the optimal clock model, we evaluated both strict and optimized relaxed clocks under a MASCOT-Skyline tree prior, which accounts for fluctuating population sizes over time. Model selection was performed using a nested sampling strategy [27], with marginal likelihoods compared to determine the best-fit combination for our dataset. For the migration rate priors, an exponential distribution (mean=0.001) was applied, while other parameters were maintained at default settings. The MCMC analysis consisted of three independent runs, each spanning 1×10^7^ iterations with sampling every 1,000 steps. Chain convergence and parameter stability were verified using Tracer 1.7.1 [28], ensuring all ESS values exceeded 200 after discarding the initial 10% burn-in.

### Genetic diversity and population genetic analysis

To determine the population structure without a priori geographic assumptions as described by Melo *et al*. [29], a discriminant analysis of principal components (DAPC) was performed using the R package *adegenet*. The optimal number of genetic clusters (*K*) was identified at the 'elbow' of the Bayesian information criterion (BIC) curve to avoid overfitting [30]. Haplotype diversity and nucleotide diversity (*π*) were estimated for the total CP dataset and each inferred subpopulation using DnaSP 6.0 [31]. To properly compare the nucleotide diversities as recommended by Lima *et al*. [32], *Z*-tests were performed utilizing the *π* estimates and their sd. Genetic differentiation among the inferred populations was quantified via pairwise *F*_ST_ values computed in Arlequin 3.5 [33].

## Results

### Assessment of recombination and temporal signals

To evaluate the suitability of our genomic dataset for phylodynamic analysis, we first screened the sequences for evidence of recombination. Phylogenetic network analysis revealed a distinct tree-like topology with no visible reticulation (Fig. S1). This observation was statistically supported by the PHI test (*P*=0.423) and multiple complementary detection methods implemented in RDP5. Under stringent criteria, no significant recombination events were detected across the global dataset. This high degree of phylogenetic congruence among these MCMV genomes effectively precludes the influence of reticulate evolution, thereby validating the use of a bifurcating tree model for subsequent analyses.

The temporal structure of the dataset was then evaluated through root-to-tip regression analysis and date-randomization tests (Fig. S2). Exploratory root-to-tip regression analysis revealed a positive correlation between genetic divergence and sampling time (*r*=0.44, *P*<1×10^−10^), although the proportion of variance explained was modest (R²=0.198). This moderate temporal association, together with the apparent variation among lineages, suggested that evolutionary rates might not be adequately described by a strict molecular clock and, therefore, warranted further formal model comparison. Because non-random sampling across genetically differentiated populations can generate spurious temporal signals, we additionally assessed the association between genetic and temporal structures using a Mantel test. A significant correlation was detected in the unclustered dataset (*r*=0.288, *P*=0.015), indicating potential confounding between population structure and sampling time. We, therefore, applied a cluster-based date-randomization test to account for this effect. The substitution-rate estimate obtained from the observed dataset was clearly separated from those generated from the clustered date-randomized datasets (Fig. S2), supporting the presence of sufficient temporal signal for molecular-clock dating.

### Phylodynamic reconstruction of global MCMV evolution and dispersal

**Fig. 1.**
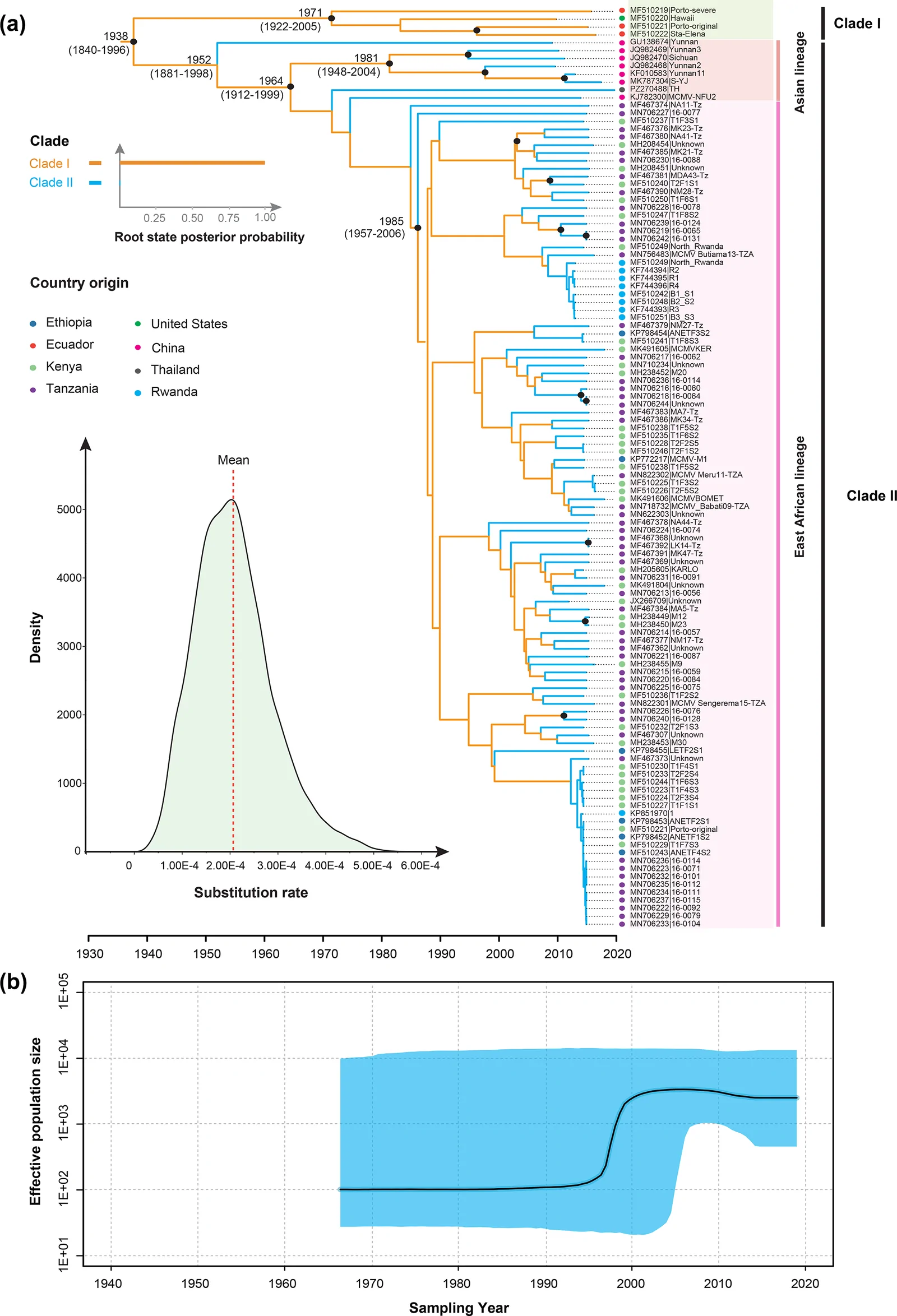
Global phylodynamics of MCMV. (**a**) Maximum clade credibility tree reconstructed from 117 recombination-free MCMV isolates using the MASCOT-Skyline framework. The tree topology represents the ensemble of the maximize the product of node posterior probabilities. Branches are colour-coded according to their cluster origins (as defined by previous phylogenetic network analysis), while coloured solid circles at the tips denote the country origin of each isolate. The red dashed line in the inset panel indicates the inferred mean substitution rate of MCMV genome. (**b**) The global population dynamics of MCMV inferred from 117 recombination-free MCMV whole-genome sequences. The *y*-axis shows the estimated effective population size (*N*_e_) on a logarithmic scale, plotted against calendar years on the *x*-axis. The shaded areas represent the corresponding 95% highest posterior density (HPD) intervals.

Model comparison using nested sampling indicated that the combination of an uncorrected relaxed clock model and the MASCOT-Skyline tree prior provided the optimal fit for the dataset, yielding a higher marginal likelihood (−12255.28) than the strict clock counterpart (−12318.22). Utilizing this validated analytical framework, Bayesian phylodynamic analysis of 117 whole-genome sequences yielded a mean substitution rate of 2.07×10^−4^ substitutions/site/year (95% CI: 6.23×10^−5^–3.69×10^−4^). The time to the most recent common ancestor (tMRCA) of global MCMV populations was estimated at 1938 (95% CI: 1840–1996). Ancestral state reconstruction provided strong statistical support for an American origin of the global population, with the root state posterior probability approaching unity (≈1) for the Americas (Fig. 1a).

Time-calibrated maximum clade credibility tree resolved a clear bipartite structure of global MCMV diversity, with the population organized into two distinct major clades (Fig. 1a). Clade I comprises basal lineages that remain predominantly restricted to the Americas, while Clade II represents a more recently derived lineage that emerged around 1952 (95% CI: 1881–1998) and is primarily composed of Asian and African isolates. The topology of Clade II exhibits a characteristic ladder-like structure, which is consistent with limited deep phylogenetic branching and recent rapid diversification patterns.

Within Clade II, the East African lineage forms a well-supported monophyletic cluster that accounts for a substantial proportion of the globally sampled isolates, highlighting its considerable epidemiological significance within our dataset. Notably, this East African lineage is phylogenetically nested within a broader assemblage of predominantly Asian isolates, suggesting patterns of regional diversification following initial establishment within Clade II. Molecular dating analysis estimated the tMRCA of the East African lineage to the mid-1980s (95% CP: 1982–1987), indicating relatively recent regional establishment.

Skyline plot reconstruction of effective population size (*N*_e_) dynamics revealed distinct demographic phases in global MCMV evolution (Fig. 1b). The analysis identified a pronounced demographic expansion phase beginning ∼1995, which reached peak intensity during the mid-2000s before subsequently stabilizing. This temporal *N*_e_ trajectory suggests a transition from a period of rapid epidemic expansion to more stable endemic transmission dynamics. The timing of this demographic expansion phase exhibits broad temporal concordance with both the diversification period of Clade II lineages and the regional establishment of the East African population, suggesting coordinated global expansion dynamics.

Collectively, these phylodynamic results provide evidence for an American origin of MCMV, followed by multiple independent intercontinental dispersal events and subsequent regional diversification. The observed patterns suggest that recent expansion phases have been particularly pronounced in Africa and Asia during the late twentieth century, coinciding with increased global agricultural connectivity and trade networks.

### Population genetic structure and signatures of recent expansion in East Africa

**Fig. 2.**
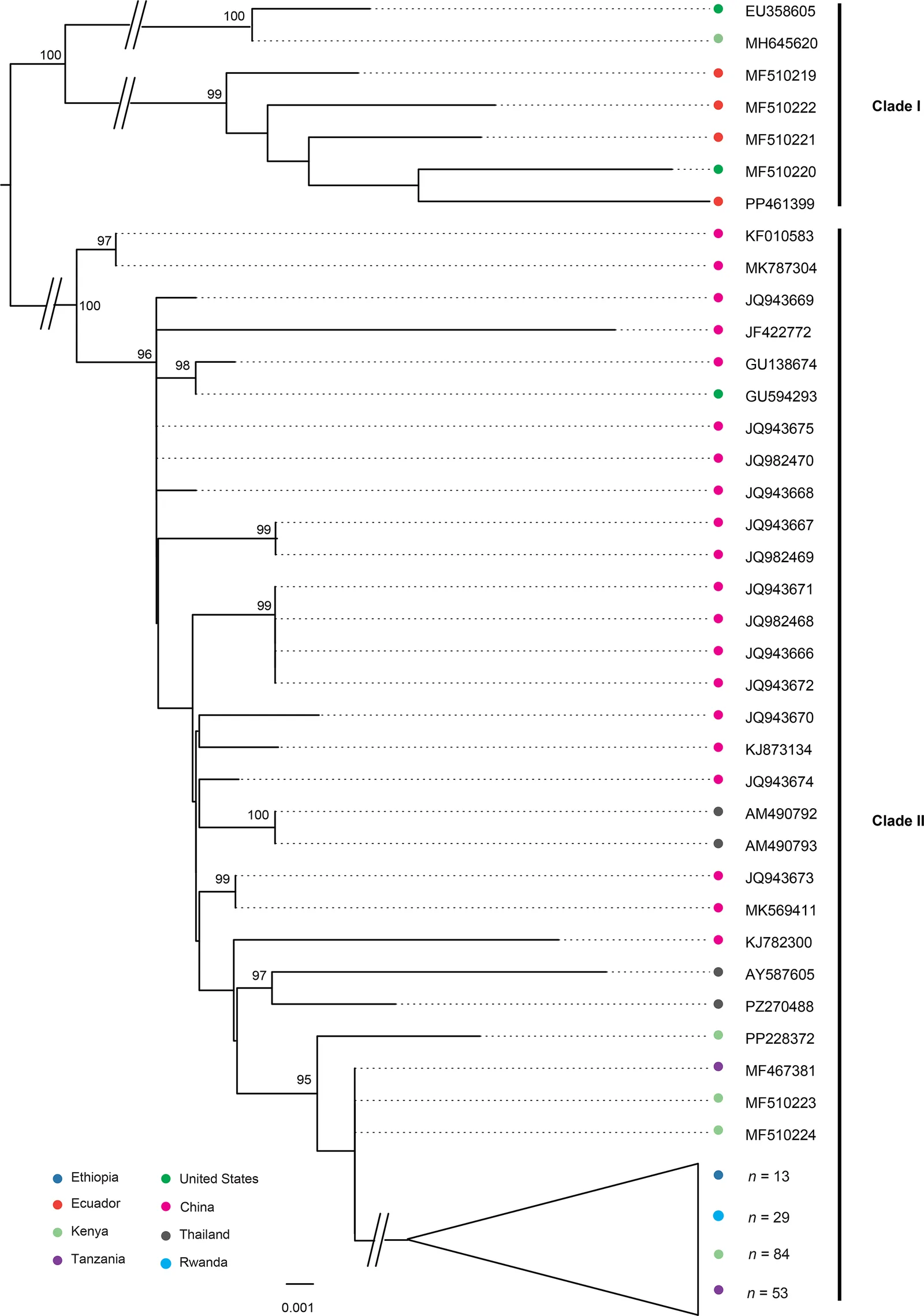
Maximum-likelihood phylogenetic tree of 214 MCMV isolates based on the CP nucleotide sequences. Ultrafast bootstrap support values >90% are indicated at key nodes. The scale bar represents the number of nucleotide substitutions per site.

To validate the robustness of our phylodynamic inferences and provide enhanced resolution of population genetic patterns, we conducted complementary analyses using an expanded CP gene dataset encompassing broader geographic sampling coverage. The CP-based phylogenetic reconstruction demonstrated remarkable consistency with whole-genome inference (Fig. 2), recovering identical major geographic clustering patterns and providing strong support for the reliability of our inferred global phylogeographic structure. The expanded dataset revealed clear genetic differentiation among American, Asian and African populations, with minimal evidence of cross-regional placement events. Notable exceptions included one African isolate clustering within Clade I and one American isolate positioned within Clade II (Fig. 2), which likely represent either sampling artefacts or rare long-distance dispersal events.

To determine whether the observed structure could be recovered without imposing predefined geographic groups, we performed DAPC. The BIC curve showed a pronounced elbow at (*K*=3), beyond which further reductions in BIC were limited (Fig. S3a). Accordingly, *K*=3 was selected as the most parsimonious representation of the major genetic clusters. The three clusters corresponded closely to the principal geographic groupings identified in the phylogenetic analyses – namely, the Americas, Asia and East Africa (Fig. S3b) – providing additional support for a geographically structured global MCMV population.

Population genetic analyses based on these inferred groups revealed substantial differences in genetic diversity (Table 1). The total population exhibited an overall nucleotide diversity of 0.007±0.0009. Among the subpopulations, the East African cluster exhibited markedly and significantly reduced nucleotide diversity (*π*=0.003±0.0004) compared with both Asian (*π*=0.008±0.0015, *Z*=3.22, *P*<0.01) and American (*π*=0.026±0.0042, *Z*=5.45, *P*<0.01) populations, representing an approximately threefold and eightfold reduction, respectively. This pattern was accompanied by consistently low pairwise *F*_ST_ values among East African countries (mean *F*_ST_=0.08), indicating minimal genetic structuring within the region. In contrast, substantially elevated *F*_ST_ values between continental populations (intercontinental mean *F*_ST_=0.68) provided strong statistical support for pronounced intercontinental genetic differentiation.

**Table 1.** Genetic diversity parameters estimated for MCMV population and pairwise *F*_ST_ values between them

Population	Sample size	Haplotype diversity	Nucleotide diversity (π)
(a)			
Clade I	7	1.000±0.0058	0.026±0.0042
Clade II	207	0.830±0.0280	0.005±0.0005
Subclade IIa	184	0.786±0.0011	0.003±0.0004
Subclade IIb	23	0.957±0.0007	0.008±0.0015
Total	214	0.841±0.0260	0.007±0.0009

(b)	Asia	America	Tanzania	Rwanda	Kenya	Ethiopia
Asia						
America	0.548 ^***^					
Tanzania	0.596 ^***^	0.815 ^***^				
Rwanda	0.561 ^***^	0.765 ^***^	0.051 ^***^			
Kenya	0.604 ^***^	0.830 ^***^	0.006 ^*^	0.066 ^***^		
Ethiopia	0.462 ^***^	0.673 ^***^	0.085 ^***^	0.136 ^***^	0.075 ^**^	

*0.01<*P*<0.05, **0.001<*P*<0.01. ***P<0.001).

These population genetic patterns collectively indicate the presence of a genetically homogeneous East African population embedded within a highly structured global framework. This signature is strongly consistent with a recent founder-driven expansion event followed by limited subsequent genetic diversification, supporting our phylodynamic reconstruction of recent regional establishment and rapid demographic expansion.

## Discussion

This study represents the most comprehensive reconstruction to date of the global molecular epidemiology of MCMV, integrating whole-genome phylodynamic inference with population genetics analyses based on the expanded CP gene datasets. Specifically, we integrated a newly characterized isolate intercepted from Thailand with publicly available genomes that include temporal and geographic metadata. Compared with previous studies relying predominantly on *CP* gene sequences [34–36], our combined approach leverages the broad sampling of *CP* data and the greater resolution of complete genomes to improve robustness of evolutionary inference. Our analytical approach has revealed a globally structured viral population characterized by distinct geographic partitioning, relatively recent evolutionary emergence and heterogeneous patterns of regional diversification.

Our phylogenetic reconstruction suggested that MCMV most likely originated in the Americas during the early 20th century, as indicated by the basal phylogenetic position of American lineages within Clade I and robust statistical support for an American ancestral state (posterior probability ≈1.0, Fig. 1a). The subsequent emergence of Clade II represents an evolutionary transition, suggesting intercontinental dispersal followed by sustained regional lineage diversification across Asia and Africa. This pattern aligns with earlier evidence of geographic partitioning [15]; however, our study advances this foundational observation by establishing a high-resolution temporal framework for these dispersal events through whole-genome phylodynamic inferences.

The predominantly ladder-like topology observed within Clade II provides important insights into the evolutionary processes underlying global MCMV dispersal. This phylogenetic structure, characterized by limited deep branching events, is consistent with repeated lineage turnover driven by serial founder effects – a demographic signature typically associated with episodic long-distance spatial dispersal followed by rapid local population expansion [37]. Given that natural insect vectors lack the dispersal capacity for transcontinental movement [8, 9], these phylodynamic patterns implicate anthropogenic mechanisms as the primary drivers of intercontinental founder events.

The well-documented seed-borne transmission capacity of MCMV provides a mechanistic explanation for these intercontinental dispersal patterns [6]. Our phylodynamic reconstruction strongly suggests that the international commercial seed trade represents the principal pathway facilitating these founder events, enabling rapid long-distance virus movement across biogeographic barriers [14]. However, we acknowledge that uneven geographic sampling density across regions may potentially contribute to the observed ladder-like topology, and this alternative explanation warrants consideration in interpreting the broader phylogenetic structure.

The temporal dynamics of global MCMV effective population size provide additional insights into the epidemiological history of this pathogen. The demographic expansion beginning in the mid-1990s, reaching peak intensity during the mid-2000s (Fig. 1b), suggests a shift in global transmission dynamics during this period. This expansion phase exhibits temporal concordance with the estimated diversification period of the East African lineage, indicating synchronized regional establishment and global population growth.

While these demographic patterns are consistent with increased viral dissemination during the late 20th century, multiple factors may have contributed to the observed expansion dynamics. These include changes in agricultural practices, intensification of maize cultivation systems, enhanced international trade networks and improved surveillance and detection capabilities [38]. The relative contributions of these factors to the observed demographic patterns represent important areas for future investigation.

The congruence between whole-genome phylodynamic inference and CP-based population genetic structure provides validation for the robustness of our inferred global phylogeographic patterns. Despite differences in genomic resolution and analytical approaches, both datasets consistently recover pronounced genetic differentiation among continental populations with limited evidence of extensive recent gene flow between major geographic regions. This population structure suggests that while episodic long-distance dispersal events have occurred throughout MCMV evolutionary history, such events remain relatively infrequent compared to local transmission and regional lineage persistence.

The East African population represents a clear case study in recent viral establishment and expansion [39]. The combination of reduced genetic diversity, minimal internal population structure and phylogenetic nesting within Asian lineages provides evidence for a relatively recent founder event followed by rapid regional expansion. While this pattern suggests founder-associated demographic processes, comprehensive sampling across the broader African continent and adjacent regions will be essential for fully resolving the number and timing of introduction events and elucidating subsequent regional transmission pathways.

Several limitations should be acknowledged. Phylodynamic analyses were based on fewer whole-genome sequences relative to CP datasets and expanded sampling – particularly from underrepresented regions – will improve estimates of evolutionary parameters and lineage relationships. In addition, uneven geographic sampling may influence inferences of population structure and dispersal patterns [40]. While temporal associations with agricultural change are suggestive, further interdisciplinary studies integrating agronomic, ecological and epidemiological data are required to clarify the drivers of MCMV emergence and spread.

In conclusion, our phylodynamic analysis supports an American origin of MCMV followed by multiple independent intercontinental dispersal events and subsequent regional diversification. The emergence of a genetically homogeneous East African population within a globally structured viral framework, coupled with evidence of recent demographic expansion, characterizes the dynamic evolutionary history of this economically important plant pathogen. These findings establish a critical foundation for understanding global MCMV dispersal patterns and underscore the necessity of implementing coordinated international surveillance programmes and robust phytosanitary measures to prevent further pathogen dissemination and protect global food security.

## Supplementary material

10.1099/jgv.0.002323 Supplementary Material 1.

## Abbreviations

BIC: Bayesian information criterion
CP: coat protein
DAPC: discriminant analysis of principal component
MASCOT: Marginal Approximation of the Structured COalescenT
MCMV: maize chlorotic mottle virus
MLN: maize lethal necrosis
PHI: pairwise homoplasy index
RACE: rapid amplification of cDNA ends
tMRCA: time to the most recent common ancestor
