# Supplementary material for: Phylodynamic reconstruction of global evolution and geographic structure of maize chlorotic mottle virus
Source: J Gen Virol. 2026 Sep 2;107(9):002323. doi: 10.1099/jgv.0.002323 (PMC13537746; doi:10.1099/jgv.0.002323)
Supplement: Supplementary file 1 [file jgv-107-02323-s001.pdf]

Supplementary Material

Phylodynamic reconstruction of global evolution and geographic structure of maize chlorotic mottle virus

Jianguo Shen [1,*], Yujie Jin[1,2], Shayidan Wufuer[1,2], Zhenguo Du [2], Fangluan Gao [2,*]

[1] *Fujian Key Laboratory for Technology Research of Inspection and Quarantine, Technology Center of Fuzhou Customs District, Fuzhou 350001, China.*

[2] *Institute of Plant Virology, Fujian Agriculture and Forestry University, Fuzhou 350002, PR China*

Supplementary Table S1 Primers for RT-PCR and RACE in this study

Segment	Name	Sequence (5'-3')
S1	S1_P1	AGACACGACCTTACAGTTGACTCAC
	S1_P2	GATCTGTTGCGCAATTTGTGTA
S2	S2_P1	ACCCTACCTTCAATTCACGCAT
	S2_P2	CCAGCAGGGAGAAGCGAAC
S3	S3_P1	CAAGCATACACTCCAGATGCAGA
	S3_P2	AGTTCGTTGGCAGTGGTTTTCT
S4	S4_P1	CCTTCAGGACTGTCTGACTAAACAC
	S4_P2	CACCCCAAGCCACTCTTCAA
S5	S5_P1	TCCATTGAGATCTGTCGCCA
	S5_P2	TGGCCCTGTATAGTAAGCGAGA
S6	S6_P1	TGAGCCAATTAGTGGGATATTTTG
	S6_P2	TGGATAGCCATAAATCCTTGTGTAT
S7	S7_P1	TGCCATAGACAAGATTTGGGGT
	S7_P2	GGTCTCTCATCATGGTGTATTGTGTA
S8	S8_P1	GCAGTGGAGGTAGGCAGAGTC
	S8_P2	ACCTCTTTTGCAATGATCACGTT
S9	S9_P1	TTGGCAGAAATCCCCGAAT
	S9_P2	CAGAATTTCGGACATGGAGCA
S10	S10_P1	CCGTTTATCACGAGCCATCGGTG
	S10_P2	CCCGATGCTAGTTTCCTAGC
5´-RACE	R1	CAGTCAGGGGCAAATTCCCACGTTA
	R2	CTCTCACAGGATGCAAGACGTGAGTCA
	RT1	GTCAACTTGTTTCGAGTAGACATCC
	RT2	CACAGACCATGTGGGTTGGATA
3´-RACE	F1	GGCAGCACAATCACAGATGTGGAACAC
	F2	ATCACTTGGGAAACATTCACAGCAGACA

Supplementary Table S2 Metadata and GenBank accession numbers for the full-genome (FG) sequences of 121 MCMV isolates analyzed in this study

No.	Isolate	Country	Collection date	Accession
1	Yunnan	China	2009-04	GU138674
2	Sichuan	China	2011-05	JQ982470
3	MCMV-NFU2	China	2014-01-06	KJ782300
4	S-YJ	China	2017-08-15	MK787304
5	Yunnan3	China	2010-05	JQ982469
6	Yunnan11	China	2013-01-25	KF010583
7	Yunnan2	China	2009-10	JQ982468
8	MCMV-TH	Thailand	2019-11-08	PZ270488
9	Porto-severe	Ecuador	2015-12	MF510219
10	Porto-original	Ecuador	2015-05	MF510221
11	Sta-Elena	Ecuador	2016-09	MF510222
12	MCMV-M1	Ethiopia	2014-10	KP772217
13	ANETF1S2	Ethiopia	2014-08-08	KP798452
14	ANETF2S1	Ethiopia	2014-08-09	KP798453
15	ANETF3S2	Ethiopia	2014-08-09	KP798454
16	LETF2S1	Ethiopia	2014-08-09	KP798455
17	ANETF4S2	Ethiopia	2014-08-09	MF510243
18	DSMZ PV-1208 *	Ethiopia	n/a	OL584368
19	MCMC_Kenya2012	Kenya	2012-02	JX286709
20	T1F4S3	Kenya	2014-08-07	MF510223
21	T2F3S4	Kenya	2014-08-14	MF510224
22	T1F3S2	Kenya	2016-08-07	MF510225
23	T2F5S2	Kenya	2016-08-15	MF510226
24	T1F1S1	Kenya	2014-08-06	MF510227
25	T2F2S5	Kenya	2014-08-14	MF510228
26	T1F7S3	Kenya	2014-08-08	MF510229
27	T1F4S1	Kenya	2014-08-07	MF510230
28	T1F7S1	Kenya	2014-08-08	MF510231
29	T2F1S3	Kenya	2014-08-13	MF510232
30	T2F2S4	Kenya	2014-08-14	MF510233
31	T1F7S2	Kenya	2014-08-08	MF510234
32	T1F6S2	Kenya	2014-08-08	MF510235
33	T1F2S2	Kenya	2014-08-06	MF510236
34	T1F3S1	Kenya	2014-08-07	MF510237
35	T1F5S2	Kenya	2014-08-07	MF510238
36	T2F5S1	Kenya	2014-08-15	MF510239
37	T2F1S1	Kenya	2014-08-13	MF510240
38	T1F8S3	Kenya	2014-08-08	MF510241
39	T1F6S3	Kenya	2014-08-08	MF510244

40	T1F5S3	Kenya	2014-08-07	MF510245
41	T2F1S2	Kenya	2014-08-13	MF510246
42	T1F8S2	Kenya	2014-08-08	MF510247
43	T1F6S1	Kenya	2014-08-08	MF510250
44	KARLO	Kenya	2014-07	MH205605
45	M12	Kenya	2015-06	MH238449
46	M23	Kenya	2015-06	MH238450
47	M18	Kenya	2015-06	MH238451
48	M20	Kenya	2015-06	MH238452
49	M30	Kenya	2016-04	MH238453
50	M13	Kenya	2016-03	MH238454
51	M9	Kenya	2016-03	MH238455
52	MCMVNYA	Kenya	2018-02-26	MK491604
53	MCMVKER	Kenya	2018-02-27	MK491605
54	MCMVBOMET	Kenya	2018-02-28	MK491606
55	DSMZ PV-1087 *	Kenya	n/a	OK181780
56	R3	Rwanda	2013	KF744393
57	R2	Rwanda	2013	KF744394
58	R1	Rwanda	2013	KF744395
59	R4	Rwanda	2013	KF744396
60	1	Rwanda	2014-06-12	KP851970
61	B1_S1	Rwanda	2013-03	MF510242
62	B2_S2	Rwanda	2013-03	MF510248
63	North_Rwanda	Rwanda	2013-03	MF510249
64	B3_S3	Rwanda	2013-03	MF510251
65	MCMV_Babati09-TZA	Tanzania	2016-05	MN718732
66	MCMV Butiama13-TZA	Tanzania	2016-05	MN756483
67	MCMV Sengerema15-TZA	Tanzania	2016-05	MN822301
68	MCMV Meru11-TZA	Tanzania	2016-05	MN822302
69	MCMV Babati10-TZA	Tanzania	2016-05	MN822303
70	16-0056	Tanzania	2015	MN706213
71	16-0057	Tanzania	2015	MN706214
72	16-0059	Tanzania	2015	MN706215
73	16-0060	Tanzania	2015	MN706216
74	16-0062	Tanzania	2015	MN706217
75	16-0064	Tanzania	2015	MN706218
76	16-0065	Tanzania	2015	MN706219
77	16-0084	Tanzania	2015	MN706220
78	16-0087	Tanzania	2015	MN706221
79	16-0092	Tanzania	2015	MN706222
80	16-0071	Tanzania	2015	MN706223
81	16-0074	Tanzania	2015	MN706224

82	16-0075	Tanzania	2015	MN706225
83	16-0076	Tanzania	2015	MN706226
84	16-0077	Tanzania	2015	MN706227
85	16-0078	Tanzania	2015	MN706228
86	16-0079	Tanzania	2015	MN706229
87	16-0088	Tanzania	2015	MN706230
88	16-0091	Tanzania	2015	MN706231
89	16-0101	Tanzania	2015	MN706232
90	16-0104	Tanzania	2015	MN706233
91	16-0111	Tanzania	2015	MN706234
92	16-0112	Tanzania	2015	MN706235
93	16-0114	Tanzania	2015	MN706236
94	16-0115	Tanzania	2015	MN706237
95	16-0121	Tanzania	2015	MN706238
96	16-0124	Tanzania	2015	MN706239
97	16-0128	Tanzania	2015	MN706240
98	16-0130	Tanzania	2015	MN706241
99	16-0131	Tanzania	2015	MN706242
100	NA11-Tz	Tanzania	2015-06-17	MF467374
101	NA16-Tz	Tanzania	2015-06-17	MF467375
102	NA44-Tz	Tanzania	2015-06-17	MF467378
103	NA41-Tz	Tanzania	2015-06-17	MF467380
104	MDA43-Tz	Tanzania	2015-04-27	MF467381
105	MA7-Tz	Tanzania	2015-06-22	MF467383
106	MA5-Tz	Tanzania	2015-06-22	MF467384
107	MA6-Tz	Tanzania	2015-06-22	MF467389
108	MK23-Tz	Tanzania	2015-06-18	MF467376
109	MK21-Tz	Tanzania	2015-06-18	MF467385
110	MK34-Tz	Tanzania	2015-06-18	MF467386
111	LK12-Tz	Tanzania	2015-06-24	MF467387
112	MK24-Tz	Tanzania	2015-06-18	MF467388
113	MK47-Tz	Tanzania	2015-06-18	MF467391
114	LK14-Tz	Tanzania	2015-06-24	MF467392
115	NM17-Tz	Tanzania	2015-06-19	MF467377
116	NM27-Tz	Tanzania	2015-06-19	MF467379
117	NM19-Tz	Tanzania	2015-06-19	MF467382
118	NM28-Tz	Tanzania	2015-06-19	MF467390
119	KS1 *	USA	n/a	X14736
120	Nebraska *	USA	n/a	EU358605
121	Hawaii	USA	2009-12	MF510220

*, detected recombinants; n/a, not available.

Supplementary Table S3 Metadata and GenBank accession numbers for the coat protein (CP) gene sequences of 214 MCMV isolates analyzed in this study

No	Isolate	Country	Collection date	Accession
1	Yunnan	China	2009-04	GU138674
2	Yuanjiang 2	China	2014-01	KJ873134
3	Sichuan	China	2011-05	JQ982470
4	SCH1	China	2011-05	JQ943675
5	MCMV-YJ	China	2010-07-20	JF422772
6	S-YJ	China	2017-08-15	MK787304
7	Yunnan11	China	2013-01-25	KF010583
8	Yunnan3	China	2010-05	JQ982469
9	YN10	China	2012-02	JQ943674
10	YN9	China	2012-02	JQ943673
11	YN6	China	2010-05	JQ943670
12	YN5	China	2010-05	JQ943669
13	YN4	China	2010-05	JQ943668
14	YN3	China	2010-05	JQ943667
15	Yunnan2	China	2009-10	JQ982468
16	YN8	China	2011-08	JQ943672
17	YN7	China	2011-08	JQ943671
18	YN2	China	2009-12	JQ943666
19	YJ	China	2017-08-15	MK569411
20	Sta-Elena	Ecuador	2016-09	MF510222
21	Porto-original	Ecuador	2015-05	MF510221
22	Porto-severe	Ecuador	2015-12	MF510219
23	Santa_Ana-Yellow_2021	Ecuador	2021-08	PP461399
24	MCMV-M1	Ethiopia	2014-12	KP772217
25	ANETF4S2	Ethiopia	2014-08-09	MF510243
26	DSMZ PV-1208	Ethiopia	n/a	OL584368
27	LETF2S1	Ethiopia	2014-08-09	KP798455
28	ANETF3S2	Ethiopia	2014-08-09	KP798454
29	ANETF2S1	Ethiopia	2014-08-09	KP798453
30	ANETF1S2	Ethiopia	2014-08-08	KP798452
31	Et227	Ethiopia	2015-08-21	MH286958
32	MV63	Ethiopia	2016-07-21	MH286954
33	MV79	Ethiopia	2016-07-22-	MH286955
34	Et111	Ethiopia	2015-08-20	MH286957
35	M48	Ethiopia	2017-03-02	MH286953
36	MV83	Ethiopia	2016-07-22	MH286956
37	MCMV.KCO24	Kenya	2016-07-19	MH645622
38	MCMV.KSM5	Kenya	2016-07-19	MH645621
39	MCMV.KS1	Kenya	2016-07-19	MH645620

40	T1F6S1	Kenya	2014-08-08	MF510250
41	T1F8S2	Kenya	2014-08-08	MF510247
42	T2F1S2	Kenya	2014-08-13	MF510246
43	T1F5S3	Kenya	2014-08-07	MF510245
44	T1F6S3	Kenya	2014-08-08	MF510244
45	T1F8S3	Kenya	2014-08-08	MF510241
46	T2F1S1	Kenya	2014-08-13	MF510240
47	T2F5S1	Kenya	2014-08-15	MF510239
48	T1F5S2	Kenya	2014-08-07	MF510238
49	T1F3S1	Kenya	2014-08-07	MF510237
50	T1F2S2	Kenya	2014-08-06	MF510236
51	T1F6S2	Kenya	2014-08-08	MF510235
52	T1F7S2	Kenya	2014-08-08	MF510234
53	T2F2S4	Kenya	2014-08-14	MF510233
54	T2F1S3	Kenya	2014-08-13	MF510232
55	T1F7S1	Kenya	2014-08-08	MF510231
56	T1F4S1	Kenya	2014-08-07	MF510230
57	T1F7S3	Kenya	2014-08-08	MF510229
58	T2F2S5	Kenya	2014-08-14	MF510228
59	T1F1S1	Kenya	2014-08-06	MF510227
60	T2F5S2	Kenya	2014-08-15	MF510226
61	T1F3S2	Kenya	2014-08-07	MF510225
62	T2F3S4	Kenya	2014-08-14	MF510224
63	T1F4S3	Kenya	2014-08-07	MF510223
64	KM47S4	Kenya	2022	PP228372
65	KM64S9	Kenya	2022	PP228371
66	KM68S13	Kenya	2022	PP228370
67	KM69S14	Kenya	2022	PP228369
68	KM71S16	Kenya	2022	PP228368
69	LTSA37S17	Kenya	2020	PP228367
70	ConnectedS50	Kenya	2017	PP228366
71	ConnectedS49	Kenya	2017	PP228365
72	ConnectedS48	Kenya	2017	PP228364
73	ConnectedS46	Kenya	2017	PP228363
74	ConnectedS45	Kenya	2017	PP228362
75	ConnectedS44	Kenya	2017	PP228361
76	ConnectedS43	Kenya	2017	PP228360
77	ConnectedS42	Kenya	2017	PP228359
78	ConnectedS41	Kenya	2017	PP228358
79	ConnectedS40	Kenya	2017	PP228357
80	ConnectedS39	Kenya	2017	PP228356
81	ConnectedS38	Kenya	2017	PP228355

82	ConnectedS37	Kenya	2017	PP228354
83	ConnectedS36	Kenya	2017	PP228353
84	ConnectedS34	Kenya	2017	PP228352
85	ConnectedS33	Kenya	2017	PP228351
86	ConnectedS32	Kenya	2017	PP228350
87	ConnectedS31	Kenya	2017	PP228349
88	ConnectedS30	Kenya	2017	PP228348
89	ConnectedS28	Kenya	2017	PP228347
90	ConnectedS27	Kenya	2017	PP228346
91	ConnectedS26	Kenya	2017	PP228345
92	ConnectedS25	Kenya	2017	PP228344
93	ConnectedS24	Kenya	2017	PP228343
94	ConnectedS23	Kenya	2017	PP228342
95	ConnectedS22	Kenya	2017	PP228341
96	ConnectedS19	Kenya	2017	PP228340
97	ConnectedS18	Kenya	2017	PP228339
98	ConnectedS17	Kenya	2017	PP228338
99	ConnectedS15	Kenya	2017	PP228337
100	ConnectedS14	Kenya	2017	PP228336
101	ConnectedS12	Kenya	2017	PP228335
102	ConnectedS11	Kenya	2017	PP228334
103	ConnectedS10	Kenya	2017	PP228333
104	ConnectedS9	Kenya	2017	PP228332
105	ConnectedS8	Kenya	2017	PP228331
106	ConnectedS7	Kenya	2017	PP228330
107	ConnectedS6	Kenya	2017	PP228329
108	ConnectedS5	Kenya	2017	PP228328
109	ConnectedS4	Kenya	2017	PP228327
110	ConnectedS2	Kenya	2017	PP228326
111	ConnectedS1	Kenya	2017	PP228325
112	MCMVBOMET	Kenya	2018-02-28	MK491606
113	MCMVKER	Kenya	2018-02-27	MK491605
114	MCMVNYA	Kenya	2018-02-26	MK491604
115	M9	Kenya	2016-03	MH238455
116	M13	Kenya	2016-03	MH238454
117	M30	Kenya	2016-04	MH238453
118	M20	Kenya	2015-06	MH238452
119	M18	Kenya	2015-06	MH238451
120	M23	Kenya	2015-06	MH238450
121	M12	Kenya	2015-07	MH238449
122	KARLO	Kenya	2014-07	MH205605
123	n/a	Kenya	2012-02	JX286709

124	DSMZ PV-1087	Kenya	n/a	OK181780
125	B3_S3	Rwanda	2013-03	MF510251
126	North_Rwanda	Rwanda	2013-03	MF510249
127	B2_S2	Rwanda	2013-03	MF510248
128	B1_S1	Rwanda	2013-03	MF510242
129	RwS15_L005	Rwanda	2015	MK684216
130	RwS14_L001	Rwanda	2015	MK684215
131	RwS11_L007	Rwanda	2015	MK684214
132	RwS07_L017	Rwanda	2015	MK684213
133	RwS03_L010	Rwanda	2015	MK684212
134	RwS02_L009	Rwanda	2015	MK684211
135	RwS03_L006	Rwanda	2015	MK684210
136	RwS02_L004	Rwanda	2015	MK684209
137	RwS02_L001	Rwanda	2015	MK684208
138	RwS01_L004	Rwanda	2015	MK684207
139	RwS01_L003	Rwanda	2015	MK684206
140	RwS01_L002	Rwanda	2015	MK684205
141	RwS01_L001	Rwanda	2015	MK684204
142	Rw-CP424	Rwanda	2015	MK684203
143	Rw-CP208	Rwanda	2015	MK684202
144	Rw-CP207	Rwanda	2015	MK684201
145	Rw-CP145	Rwanda	2015	MK684200
146	Rw-CP43	Rwanda	2015	MK684199
147	Rw-CP18	Rwanda	2015	MK684198
148	Rw-CP01	Rwanda	2015	MK684197
149	1	Rwanda	2014-06-12	KP851970
150	R4	Rwanda	2013	KF744396
151	R1	Rwanda	2013	KF744395
152	R2	Rwanda	2013	KF744394
153	R3	Rwanda	2013	KF744393
154	MCMV-NFU2	Taiwan	2014-01-06	KJ782300
155	MCMV Butiama13-TZA	Tanzania	2016-05	MN756483
156	MCMV Babati10-TZA	Tanzania	2016-05	MN822303
157	MCMV Meru11-TZA	Tanzania	2016-05	MN822302
158	MCMV Sengerema15-TZA	Tanzania	2016-05	MN822301
159	MCMV_Babati09-TZA	Tanzania	2016-05	MN718732
160	16-0131	Tanzania	2015	MN706242
161	16-0130	Tanzania	2015	MN706241
162	16-0128	Tanzania	2015	MN706240
163	16-0124	Tanzania	2015	MN706239
164	16-0121	Tanzania	2015	MN706238
165	16-0115	Tanzania	2015	MN706237

166	16-0114	Tanzania	2015	MN706236
167	16-0112	Tanzania	2015	MN706235
168	16-0111	Tanzania	2015	MN706234
169	16-0104	Tanzania	2015	MN706233
170	16-0101	Tanzania	2015	MN706232
171	16-0091	Tanzania	2015	MN706231
172	16-0088	Tanzania	2015	MN706230
173	16-0079	Tanzania	2015	MN706229
174	16-0078	Tanzania	2015	MN706228
175	16-0077	Tanzania	2015	MN706227
176	16-0076	Tanzania	2015	MN706226
177	16-0075	Tanzania	2015	MN706225
178	16-0074	Tanzania	2015	MN706224
179	16-0071	Tanzania	2015	MN706223
180	16-0092	Tanzania	2015	MN706222
181	16-0087	Tanzania	2015	MN706221
182	16-0084	Tanzania	2015	MN706220
183	16-0065	Tanzania	2015	MN706219
184	16-0064	Tanzania	2015	MN706218
185	16-0062	Tanzania	2015	MN706217
186	16-0060	Tanzania	2015	MN706216
187	16-0059	Tanzania	2015	MN706215
188	16-0057	Tanzania	2015	MN706214
189	16-0056	Tanzania	2015	MN706213
190	MA6-Tz	Tanzania	2015-06-22	MF467389
191	MA5-Tz	Tanzania	2015-06-22	MF467384
192	MA7-Tz	Tanzania	2015-06-22	MF467383
193	MDA43-Tz	Tanzania	2015-04-27	MF467381
194	NA41-Tz	Tanzania	2015-06-17	MF467380
195	NA44-Tz	Tanzania	2015-06-17	MF467378
196	NA16-Tz	Tanzania	2015-06-17	MF467375
197	NA11-Tz	Tanzania	2015-06-17	MF467374
198	LK14-Tz	Tanzania	2015-06-24	MF467392
199	MK47-Tz	Tanzania	2015-06-18	MF467391
200	MK24-Tz	Tanzania	2015-06-18	MF467388
201	LK12-Tz	Tanzania	2015-06-24	MF467387
202	MK34-Tz	Tanzania	2015-06-19	MF467386
203	MK21-Tz	Tanzania	2015-06-18	MF467385
204	MK23-Tz	Tanzania	2015-06-18	MF467376
205	NM28-Tz	Tanzania	2015-06-19	MF467390
206	NM19-Tz	Tanzania	2015-06-19	MF467382
207	NM27-Tz	Tanzania	2015-06-19	MF467379

208	NM17-Tz	Tanzania	2015-06-19	MF467377
209	CP	Thailand	N/A	AY587605
210	MC14-2	Thailand	N/A	AM490793
211	MC3/1-15	Thailand	N/A	AM490792
212	MCMV-us	USA	2009-09	GU594293
213	Hawaii	USA	2009-12	MF510220
214	Nebraska	USA	N/A	EU358605

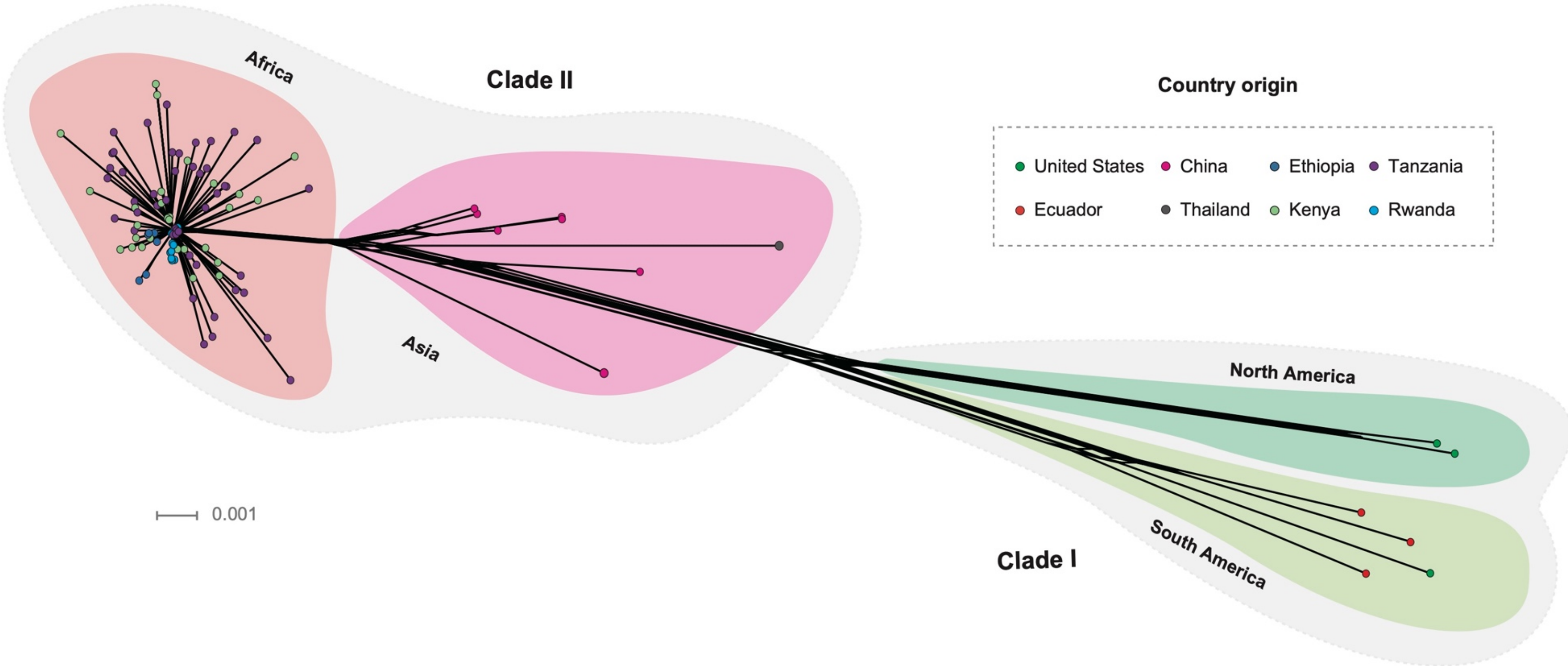

Supplementary Fig. S1 Phylogenetic networks of 121 MCMV isolates based on the complete genome sequences. MCMV isolates from different countries are indicated by a unique color. Branch lengths are proportional to the genetic distances

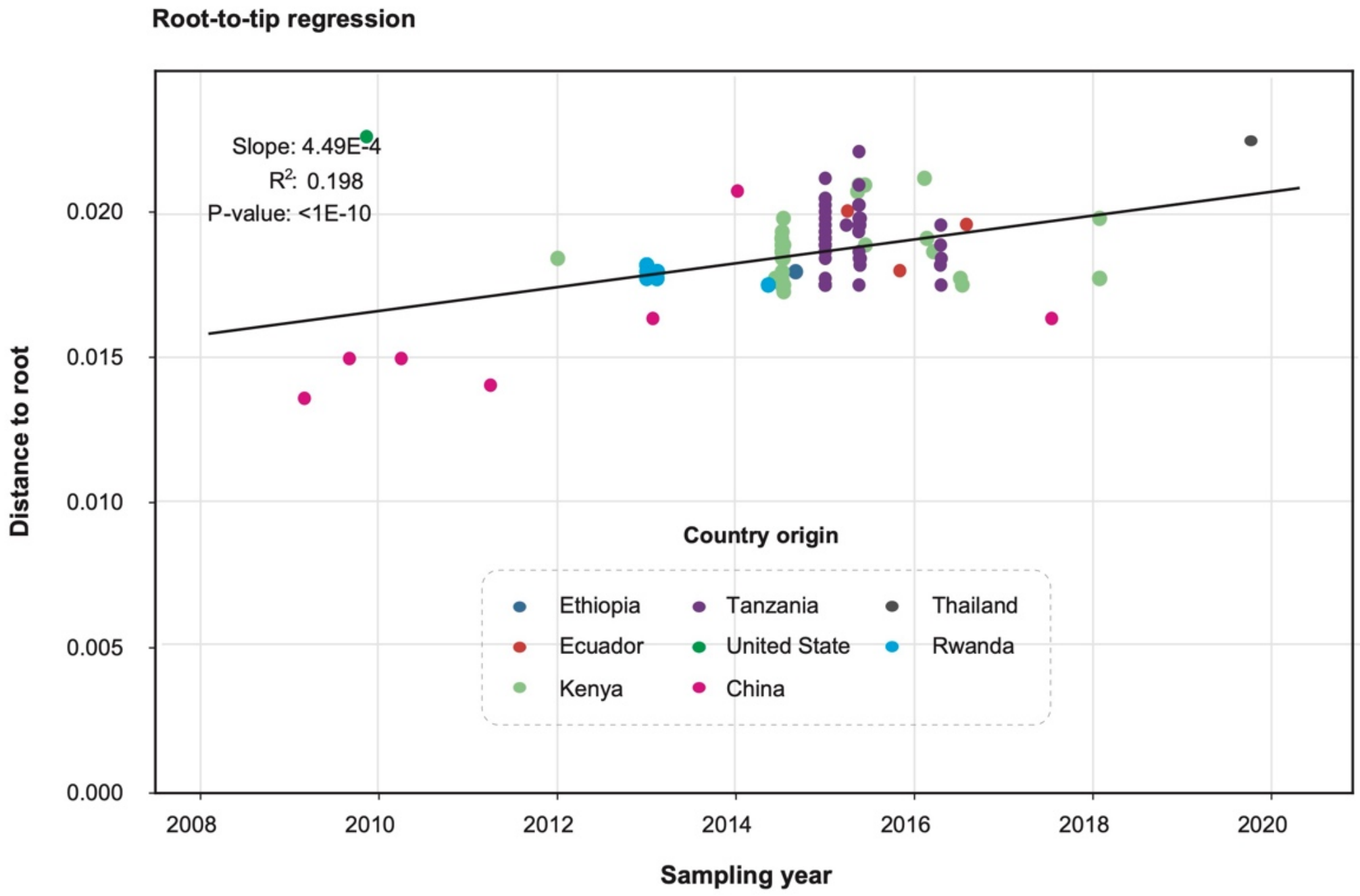

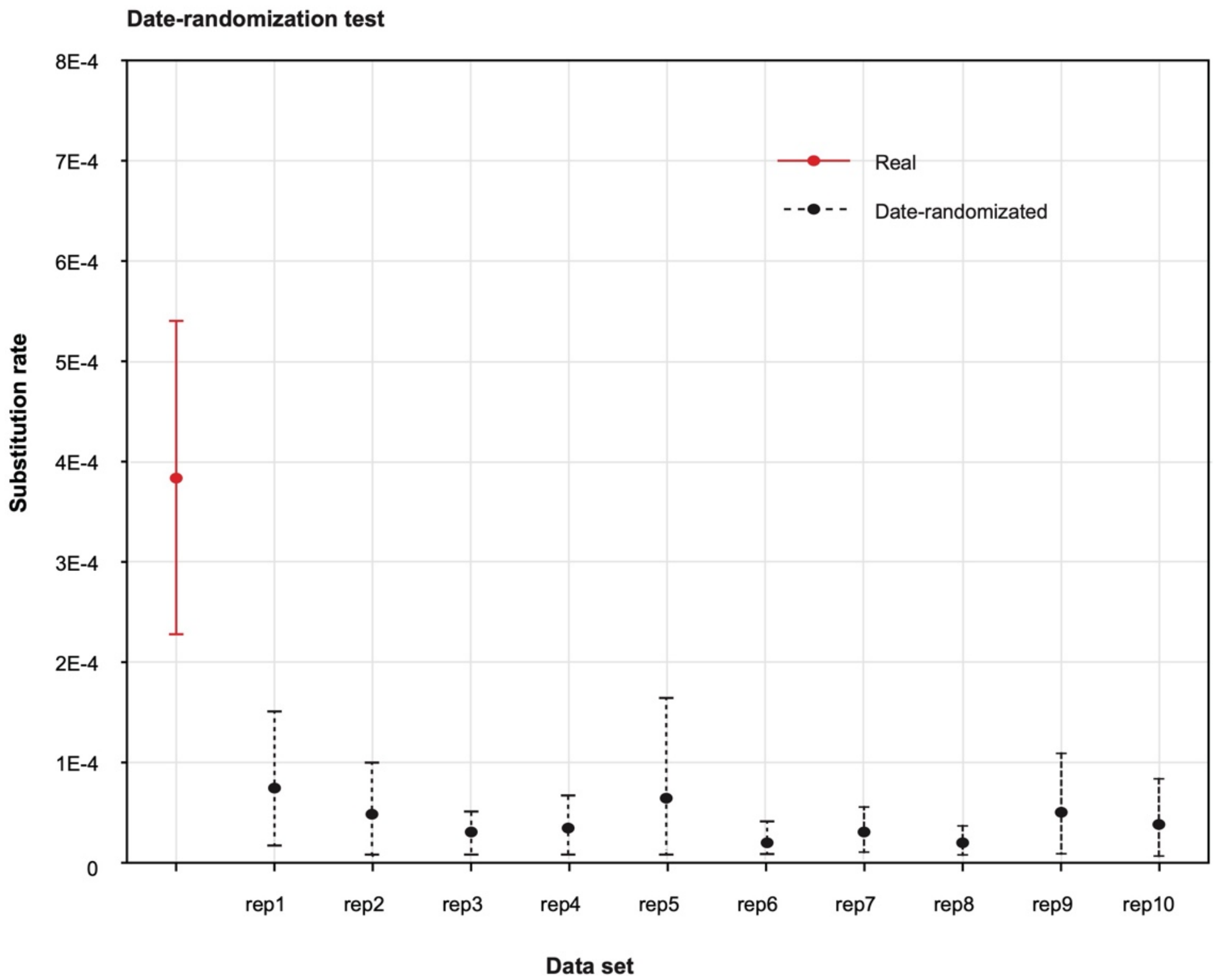

Supplementary Fig. S2 Assessment the temporal signal in the sequence data using a root-to-tip regression and a clustered date-randomization test. Viral isolates from different countries are indicated by a unique color. Substitution rate estimates for the original dataset are plotted alongside 10 randomized replicates. Vertical bars indicate the 95% credibility intervals (red solid line: real data; black dash line: randomized replicates)

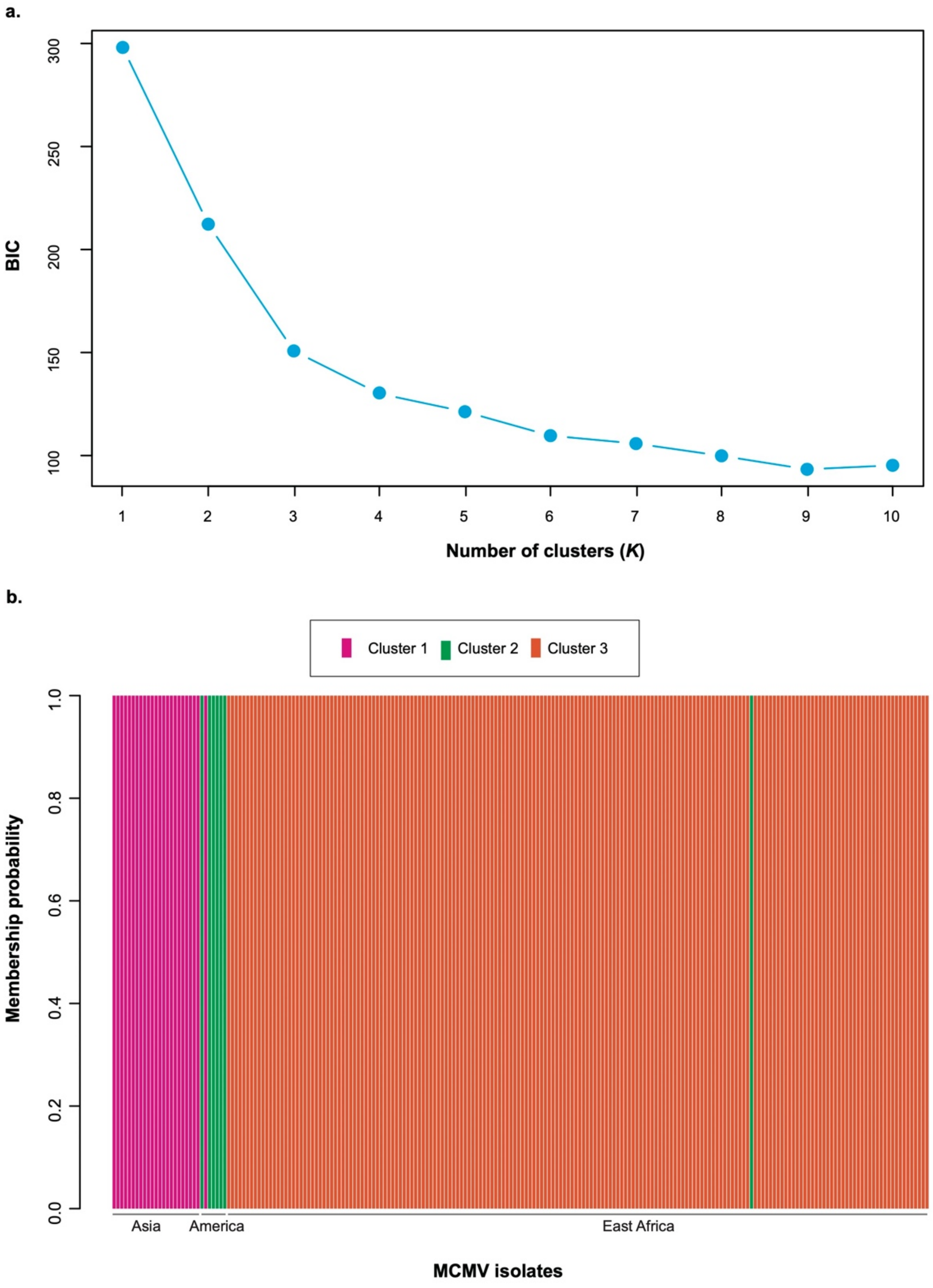

Supplementary Fig. S3 Discriminant analysis of principal components (DAPC) of the MCMV population. (a) Bayesian information criterion (BIC) values used to infer the optimal number of genetic clusters (*K*). (b) Membership probability of individual isolates assigned to the inferred clusters based on the CP gene dataset
